# Effects of Replacing Soybean Meal with Different Proportions of Black Soldier Fly Larvae Meal on Antioxidant Indicators, Immune System, and Gut Health of Xichuan Black-Bone Chickens

**DOI:** 10.3390/antiox15040408

**Published:** 2026-03-24

**Authors:** Xiaowen Geng, Luyu Yang, Yingdong Hou, Zhiyuan Zhang, Fumin He, Ruilong Xu, Pengwei Zhang, Ruirui Jiang, Wenting Li, Guirong Sun, Xiaojun Liu, Ruili Han, Xiangtao Kang, Yadong Tian, Donghua Li

**Affiliations:** 1 College of Animal Science and Technology, Henan Agricultural University, Zhengzhou 450046, China; gengxiaowen2022@163.com (X.G.); yly24580@163.com (L.Y.);; 2 Key Laboratory of Livestock and Poultry Resources (Poultry) Evaluation and Utilization of Ministry of Agriculture and Rural Affairs, Henan Agricultural University, Zhengzhou 450046, China

**Keywords:** black soldier fly larvae meal, feed substitution, antioxidant capacity, intestinal microbiota, metabolic regulation

## Abstract

This study aimed to investigate the underlying mechanisms by which replacing soybean meal with different proportions of black soldier fly (BSF) larvae meal affects the antioxidant capacity, immune function, and intestinal health in Xichuan black-bone chickens. After feeding the diets with 0.0%, 3.9%, 7.8%, 11.7%, and 15.6% BSF powder for 56 days, twelve chickens were sampled from each group. The optimal addition group was determined. Compared with the control group, adding 11.7% BSF significantly increased serum T-AOC, SOD, IgA, IgM, IL-4, and IL-10 concentrations, while markedly reducing IL-1β, TNF-α, and IL-6 (*p* < 0.05) and improving spleen morphology. Adding 11.7% BSF significantly increased the duodenal villus-to-crypt ratio (V/C) and markedly elevated the ileal V/C (*p* < 0.05), and it significantly increased gene expression levels of duodenal *Claudin-1*, *Occludin*, and *E-cadherin*, jejunal *Claudin-1*, and ileal *Claudin-1*, *Occludin*, and *E-cadherin* (*p* < 0.05). Compared with the control group, the 11.7% addition group exhibited significant alterations in caecal microbiota composition (*p* < 0.05), with 10 distinct bacterial genera identified at the genus level. A total of 424 differentially expressed metabolites were identified. Correlation analyses revealed that adding 11.7% BSF may enhance immune function by regulating intestinal metabolites such as isovaleric acid, inosine, and tazarotenic acid via *Akkermansia, Sphaerochaeta,* and *Blautia* in the cecum. It may also improve gut health by modulating inosine through *Sphaerochaeta* and *Blautia*. This trial provides feasibility evidence for substituting soybean meal with BSF meal, offering scientific support for sustainable development in animal husbandry.

## 1. Background

With the rapid expansion of the poultry industry, the demand for protein feed resources continues to grow, making the search for sustainable protein feed ingredients a focal point for the sector. As a traditional protein feed, soybean meal has long held a significant position in livestock, poultry, and aquaculture [1]. However, issues such as price volatility and resource scarcity have become increasingly prominent, posing major constraints to the sustainable development of the feed industry [2]. Furthermore, soybean meal, as a high-protein dietary component, generates substantial nitrogen and phosphorus emissions during cultivation, exacerbating water eutrophication and soil pollution [3]. While it is currently the primary protein source in poultry feed and exhibits high utilization efficiency by chickens, its inherent limitations and environmental pressures necessitate the urgent development of alternative protein sources within the feed industry. Insect protein, as a novel sustainable protein resource (such as insect proteins from yellow mealworm and black soldier fly), is witnessing continuous expansion in its feed market scale. The black soldier fly, emerging as an innovative feed ingredient, possesses the characteristic of efficiently converting organic waste into high-protein feed. Its rapid growth rate, strong reproductive capacity, and potential for green circular production make it an ideal alternative to traditional proteins [4]. Adding 9% black soldier fly larvae meal can improve the nutrient digestibility of 1-day-old broilers, replacing 50% of soybean meal can improve the feed conversion rate of quails, and the complete replacement of fish meal can promote the expression of antimicrobial peptides in weaned piglets [5,6,7]. Utilizing livestock manure for black soldier fly cultivation not only reduces production costs but also promotes resource recycling. BSFs are rich in essential amino acids for poultry, such as lysine and methionine, making them an excellent material for developing protein feed. BSF meal boasts high crude protein content alongside essential amino acids and bioactive components (e.g., chitin and lauric acid), demonstrating significant advantages in enhancing animal growth performance [8]. It may also affect the antioxidant status, serum immunity, and intestinal physiology of poultry.

As a precious local breed in China serving dual purposes for meat, eggs, and medicinal use, Xichuan black-bone chickens possess distinct genetic characteristics and economic value [9]. However, their large-scale farming faces multiple constraints. Against the backdrop of antibiotic prohibition, the antioxidant function and immune function of breeding cocks decline with age, shortening their productive lifespan and potentially compromising conservation efforts [10]. Furthermore, intestinal diseases remain a significant challenge in large-scale farming of local chicken breeds.

Antioxidant indicators can evaluate the ability of poultry organisms to protect cells from oxidative damage and maintain physiological functions [11]. Immune function defends against pathogenic infections, while the gut serves as a vital organ for nutrient absorption [12]. These three indicators all reflect the overall health of the animals. Antioxidant capacity is usually determined by SOD, T-AOC, MDA, and GSH. Immune parameters include spleen morphology, serum immunoglobulin levels, and inflammatory factor concentrations. Gut health is typically assessed through villus and crypt morphology, villus-to-crypt depth ratios, and intestinal connectin levels. Existing research has primarily focused solely on the effects of black soldier flies (BSFs) on growth performance (such as daily weight gain and feed conversion ratio) [13]. Investigation into the cascade regulatory mechanism by which black soldier flies modulate antioxidant capacity and other indicators through the pathway ‘dietary intake–gut microbiota–intestinal metabolites–organism’ and systematic analyses of local breeds (such as Xichuan black-bone chickens) are lacking; moreover, the analysis of their efficacy primarily relies on single-omics approaches. Integrating multi-omics analyses (e.g., 16S rRNA gene amplicon sequencing, metagenomics, and metabolomics) to elucidate BSF’s action mechanisms in local breeds is therefore particularly crucial.

To investigate the optimal inclusion rate of BSF in Xichuan black-bone chickens and its action mechanisms, this trial employed four inclusion rates of BSF (3.9%, 7.8%, 11.7%, and 15.6%) to replace soybean meal on a one-to-one basis. It also evaluated the effects of black soldier fly meal on antioxidant indicators, immune function, and gut health in Xichuan black-bone breeding cocks, identifying the optimal supplementation group. The gut microbiota of the optimal supplementation group and the control group was analyzed via 16S rRNA sequencing. The cecum, exhibiting the most significant changes in microbial community distribution and the highest Shannon index, was selected for analysis of deeper microbial communities and metabolites. Finally, differential microbes, metabolites, and indicators were correlated to predict the mechanism by which BSF meal influences physiological status through gut microbiota and metabolites.

## 2. Materials and Methods

The protocol of this experiment was reviewed and approved by the Animal Care and Use Committee of Henan Agricultural University.

### 2.1. Animals, Experimental Design, and Experimental Diets

A total of 120 healthy, similarly sized 470-day-old Xichuan black-boned breed roosters (uniform body weight) were randomly divided into five groups (0%, 3.9%, 7.8%, 11.7%, and 15.6% BSF meal supplementation groups), with six replicates per group and four chickens per replicate. The pre-trial period lasted 14 days, followed by a 56-day experimental period. BSFs were supplied by Zhengzhou Kuocheng Ecological Agriculture Technology Co., Ltd. (Zhengzhou, China), and they were microwaved, dried, and pulverized. Their nutritional composition is detailed in Table 1. Through minor adjustments to other nutrients, diets across groups were standardized for nitrogen, energy, and essential amino acid content. Diet composition and nutritional levels are outlined in Table 2. The experiment was conducted at Xingshengyuan Animal Husbandry Co., Ltd. in Xichuan County, China, strictly adhering to animal welfare and ethical standards. Throughout the feeding period, chickens had free access to feed and water. Disinfection and semen collection were performed in accordance with the routine requirements of the chicken farm. Environmental temperature, humidity, and lighting were controlled according to breeding rooster rearing standards.

### 2.2. Sample and Data Collection

At the end of the experiment, two chickens were randomly selected from each replicate, with 12 chickens per group used for sample collection. First, blood was collected via venipuncture to prepare serum, which was stored at −80 °C. The chickens were then euthanized, and the entire spleen as well as the middle third (5 cm each) of the duodenum, jejunum, and ileum were collected and placed in formalin fixative. A 1.5 cm intestinal tissue segment was taken from the middle of each intestinal section; molecular samples of the duodenum, jejunum, and ileum tissues were rinsed with 0.9% saline solution, placed in sterile, enzyme-free centrifuge tubes, and stored at −80 °C [14]. Twelve chickens were selected from the control group and each of the different black soldier fly (BSF) supplementation groups. Intestinal contents from two birds per replicate were pooled and homogenized for subsequent microbial and metabolite analyses. Based on the results of antioxidant indices, immune indices, and intestinal physiological morphology indices, the optimal BSF supplementation group was determined. Subsequently, 16S rRNA gene sequencing was performed to analyze microbial communities in different intestinal segments. According to changes in microbial diversity and community distribution, notable microbial communities in specific intestinal segments were selected for metagenomic sequencing. Using the same sample collection and processing method, intestinal contents from the notable segments were collected from the control group and the optimal supplementation group for metabolomic analysis. After extraction with methanol/acetonitrile, the supernatants were collected for mass spectrometry (MS) detection.

### 2.3. Serum-Related Indicators

Serum concentrations of Total Antioxidant Capacity (T-AOC), Malondialdehyde (MDA), Superoxide Dismutase (SOD), and Glutathione (GSH) were determined using kits from Nanjing Jiancheng Bioengineering Institute (Nanjing, China). Serum Immunoglobulin A (IgA), Immunoglobulin G (IgG), Immunoglobulin M (IgM), and inflammatory cytokines [Interleukin-4 (IL-4), Interleukin-10 (IL-10), Tumor Necrosis Factor-alpha (TNF-α), Interleukin-6 (IL-6), and Interleukin-1β (IL-1β)] were measured by ELISA kits from Jiangsu Enzyme Immunoassay Biotechnology Co., Ltd (Yancheng, China). For antioxidant indices (T-AOC, MDA, SOD, and GSH), serum samples were appropriately diluted with buffer solution or distilled water (e.g., 1:10 or 1:20). Kit reagents were taken out of the refrigerator and equilibrated at room temperature for 30 min, then prepared as required (e.g., chromogenic agent, substrate, and standard) with thorough mixing to avoid bubbles. Serial gradient standard solutions (e.g., 0, 5, 10, 20, 40, and 80 μmol/L) were prepared. Standards (50-100 μL per well) and diluted serum samples (50-100 μL per well) were added to 96-well plates, followed by sequential addition of chromogenic agent and substrate as instructed. The plate was incubated at 37 °C for 15-30 min, and the reaction was terminated with stop solution (sulfuric acid). OD values were measured at a specific wavelength of 520 nm using a microplate reader. For serum immunoglobulins (IgA, IgG, and IgM) and inflammatory cytokines (IL-4, IL-10, TNF-α, IL-6, and IL-1β), required strips were taken from foil bags equilibrated at room temperature for 20 min, and the rest were sealed and stored at 4 °C. Standard and sample wells were set: 50 μL standards (different concentrations) per standard well, 10 μL samples + 40 μL sample diluent per sample well, and blank wells were left empty. Except for blank wells, 100 μL Horseradish Peroxidase (HRP)-labeled detection antibody was added to each well, sealed with a plate sealer, and incubated at 37 °C for 60 min. After discarding the liquid, the plate was washed five times (filled with washing buffer, standing for 1 min, discarded and patted dry). Then 50 μL each of substrate A and B were added, incubated at 37 °C in the dark for 15 min, followed by 50 μL of stop solution. OD values were measured at 450 nm within 15 min. All indices were tested in triplicate. Standard curves were plotted with standard concentration as the abscissa and the OD value as the ordinate using Excel for linear regression, and sample concentrations were calculated via the curve equation (R^2^ ≥ 0.99). Instruments were calibrated before use to ensure result accuracy and reproducibility.

### 2.4. The Physiological Morphology of the Spleen and the Morphology of the Intestine

Spleen and intestinal tissues were removed from fixative, trimmed, and placed into labeled dehydration cassettes. Dehydration (gradient ethanol series) and paraffin infiltration were performed sequentially, followed by embedding at 65 °C, cooling at −20 °C, and trimming into 4-μm sections. Sections were flattened on 40 °C water, mounted on slides, and baked dry. HE staining included dewaxing to water, hematoxylin (2 min) and eosin (3 min) staining, dehydration, clearing, and neutral gum mounting. Sections were microscopically examined, and Motic DSAssistant Lite software (Motic DSAssistant Lite 1.0) was used to measure intestinal villus length and crypt depth. The villus-to-crypt ratio (V/C) was calculated using the formula: Villus-to-crypt ratio (V/C) = Villus length/Crypt depth.

### 2.5. Intestinal Connectin

For the gene expression quantification of intestinal connectin, total RNA was extracted from tissue using the Trizol method. Qualified RNA was reverse transcribed into cDNA following the Vazyme reverse transcription kit protocol (Nanjing Novizan Biotechnology Co., Ltd., Nanjing, China). Primers were designed using the Primer-BLAST online software (https://www.ncbi.nlm.nih.gov/tools/primer-blast/, 7 March 2023), referencing the NCBI database. Primer details are provided in Table 3. Real-time quantitative PCR was performed using 2×ChamQ Universal SYBR qPCR Master Mix according to the kit protocol. Following reaction completion, relative gene expression levels were calculated via the 2^(−ΔΔCt)^ method to analyze experimental variations.

### 2.6. Gut Microbiome Analysis

For 16S rRNA sequencing, following genomic DNA extraction from the control and the optimal BSF supplementation group samples, barcoded specific primers were employed to amplify the V3+V4 region of 16S rDNA. The primer sequences were: 341F: (CCTACGGGNGGCWGCAG) and 806R: (GGACTACHVGGGTATCTAAT). Sequencing libraries were constructed, and illumina sequencing was performed. Raw data were filtered and assembled into clean tags, from which effective tags were derived after de-chimerisation. These were used for OTU clustering and abundance statistics. Analysis proceeded sequentially through species annotation, α-diversity analysis, β-diversity analysis, and community functional prediction. Inter-group comparisons and statistical tests were conducted on effective groupings. Among them, differential abundance analysis of species between the two groups was performed using Welch’s *t*-test. Prior to the analysis, data preprocessing was conducted to filter low-abundance species: taxa with a total abundance sum lower than 0.1% across all samples were excluded to reduce background noise and ensure the reliability of differential results. The differential analysis was carried out at all taxonomic levels from phylum to species. Statistical significance was determined with a threshold of *p*-value < 0.05 [15].

For metagenomic sequencing, samples of key intestinal segment contents from the control group and the optimal BSF supplementation group were selected. Total DNA was extracted from these samples, with DNA quality assessed using methods such as agarose gel electrophoresis. Following quality control, sequencing libraries were constructed and subjected to library quality assessment. Upon passing library quality checks, sequencing was performed using the Illumina platform with a PE150 read length. Initial quality control filtering was applied to sequencing reads, with retained clean reads undergoing assembly to generate contigs (i.e., contiguous long sequences). Gene prediction was performed based on contigs, followed by redundancy removal to obtain a Unigene set. Clean reads were re-aligned against Unigenes for gene quantification. Unigene sequences were annotated functionally by alignment against databases such as KEGG and eggNOG. Analyses of alpha diversity, beta diversity, species/function distribution, and species/function differences were conducted based on species/function abundance tables.

### 2.7. Analysis of the Key Intestinal Segment Metabolites

Metabolite sequencing of the key intestinal segment was performed via metabolomics. Following the extraction of key intestinal segment contents, the supernatant was subjected to chromatography-mass spectrometry analysis. Samples were separated using an Agilent 1290 Infinity LC (Agilent Technologies, Inc., Waldbronn, Germany) ultra-high-performance liquid chromatography (UHPLC) system with a HILIC column. Throughout the analysis, samples were maintained at 4 °C within an autosampler. To mitigate the impact of instrument signal fluctuations, samples were analyzed consecutively in random order. Quality control (QC) samples were interspersed within the sample cohort to monitor and evaluate system stability and the reliability of experimental data. Primary and secondary mass spectra were then acquired using an AB TripleTOF 6600 mass spectrometer (AB Sciex LLC, Framingham, MA, USA).

Raw data from chromatography-mass spectrometry analysis were converted into MzML format using ProteoWizard (ProteoWizard Software Foundation, Palo Alto, CA, USA), followed by peak alignment, retention time correction, and peak area extraction via the XCMS program. For data extracted by XCMS, integrity checks were first performed. Metabolites with over 50% missing values within groups were excluded from subsequent analysis. Missing values were imputed using KNN, outliers were removed, and the data were finalized with total peak area normalization to ensure parallel comparability between samples and metabolites. Raw data were subjected to peak alignment and denoising. Differential metabolites were screened by OPLS-DA, annotated via KEGG, and differential pathways were identified by Fisher’s exact test [16].

### 2.8. Correlation Analysis of Microorganisms, Metabolites and Immune Markers

Based on 16S rRNA sequencing results, a Welch’s *t*-test was conducted on the key intestinal segment microbiota between the control and supplemented groups to identify significantly different microbial genera at the genus level. Microbial abundance data from distinct key intestinal segment samples were summed and ranked by average abundance, selecting the top 10 genera.

Based on metabolome sequencing results, Welch’s *t*-tests were conducted on gut metabolites between the control and supplemented groups to identify significantly different metabolites. The concentrations of these metabolites across different samples were summed, ranked by average concentration, and the top were 20 selected. Metabolites previously reported to be associated with immune function and gut health were identified and highlighted in bold within the table. The immune parameters for each replicate pair of chickens were averaged (consistent with the metabolome sequencing approach). Spearman’s correlation analysis was performed between gut microbial abundance and gut metabolite concentration values. Subsequently, Spearman’s correlation analysis was conducted between metabolites and immune indicators, with results visualized via heatmaps.

### 2.9. Data Statistics and Analysis

Outliers were excluded based on the criterion of the mean ± two standard deviations (SD). Data from three or more groups were analyzed using one-way analysis of variance (ANOVA) in SPSS 26.0. Differences between two groups were assessed using independent samples *t*-tests or non-parametric tests, with results expressed as the mean ± standard error of the mean (SEM). Results were considered statistically significant at *p* < 0.05, with 0.05 ≤ *p* ≤ 0.1 indicating a trend toward an increase or decrease. Data and graphical processing were performed using GraphPad Prism 8.0 and Adobe Illustrator 2022.

## 3. Result

### 3.1. Effects of Different Proportions of Black Soldier Fly Larvae Meal on Serum-Related Indicators

**Figure 1:**
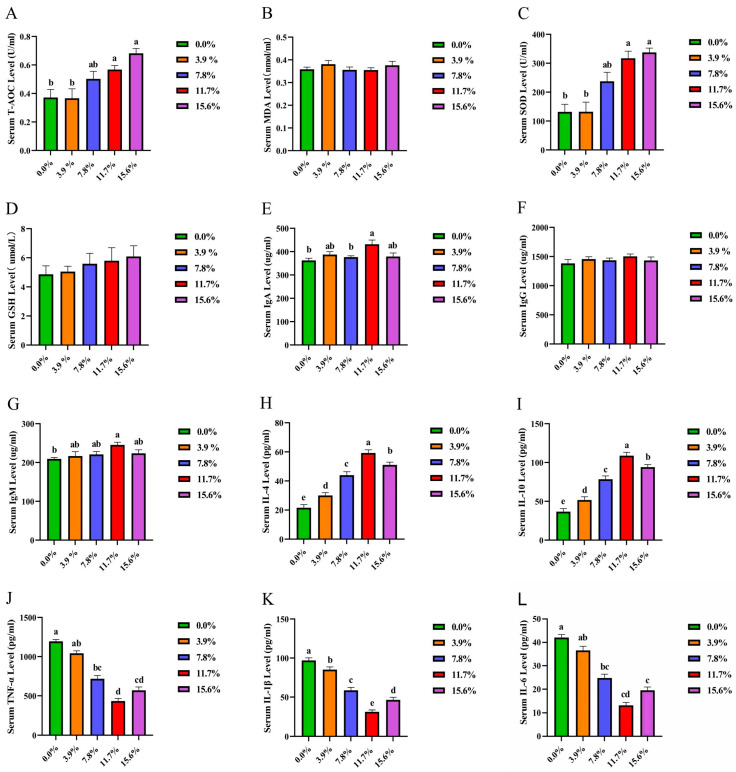
The Effect of replacing soybean meal with BSF meal on antioxidant and immunity indices of Xichuan black-bone chicken. (**A**) Serum T-AOC level; (**B**) Serum MDA level; (**C**) Serum SOD level; (**D**) Serum GSH level; (**E**) Serum IgA level; (**F**) Serum IgG level; (**G**) Serum IgM level; (**H**) Serum IL-4 level; (**I**) Serum IL-10 level; (**J**) Serum TNF-α level; (**K**) Serum IL-1β level; (**L**) Serum IL-6 level. In x-axis, the 0.0% represents the control group. The small letters a, b, etc., on the top of each bar represent statistical significance. Data with different small letters on each bar are statistically significant (*p* < 0.05), while data with the same letters are not statistically significant (*p* ≥ 0.05).

Results indicate that supplementation with 11.7% and 15.6% BSF powder significantly elevated (*p* < 0.05) serum T-AOC (Figure 1A) and SOD (Figure 1C) levels, while also enhancing serum GSH (Figure 1D). Compared with the control group, the addition of 11.7% BSF powder significantly increased (*p* < 0.05) serum IgA and IgM levels and elevated serum IgG levels (Figure 1E–G). Different proportions of BSF meal significantly increased (*p* < 0.05) serum IL-4 and IL-10 concentrations (Figure 1H,I) while markedly decreasing (*p* < 0.05) serum IL-1β levels (Figure 1K). Additions of 7.8%, 11.7%, and 15.6% BSF meal significantly reduced (*p* < 0.05) IL-6 and TNF-α concentrations (Figure 1J,L), with the 11.7% addition demonstrating the most pronounced effect on inflammatory factors.

### 3.2. Effects of Different Proportions of Black Soldier Fly Larvae Meal on the Physiological Morphology of the Spleen and the Morphology of the Intestinal Tract

**Figure 2:**
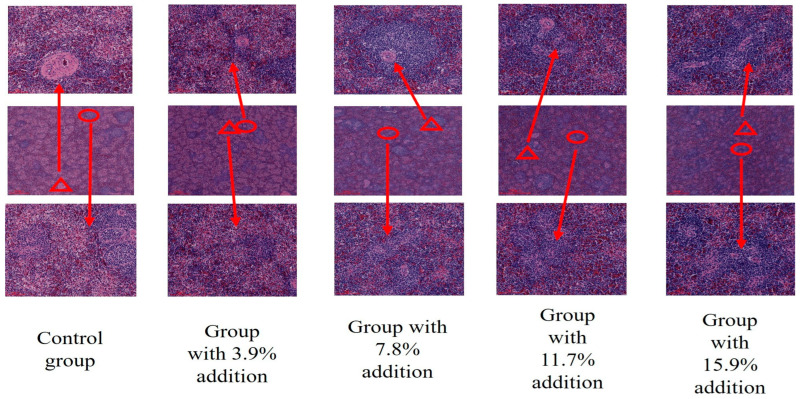
Effect of replacing soybean meal with BSF meal on the spleen morphology of Xichuan black-bone chicken. ○ indicates white pulp; △ indicates the splenic artery; top row: enlarged views of white pulp; bottom row: enlarged views of the splenic artery.

Compared with the control group, the addition of 7.8% and 11.7% BSF powder significantly improved the morphological integrity of the spleen’s white pulp, with a clear demarcation between the red and white pulp, thereby enhancing the physiological condition of the spleen (Figure 2).

**Figure 3:**
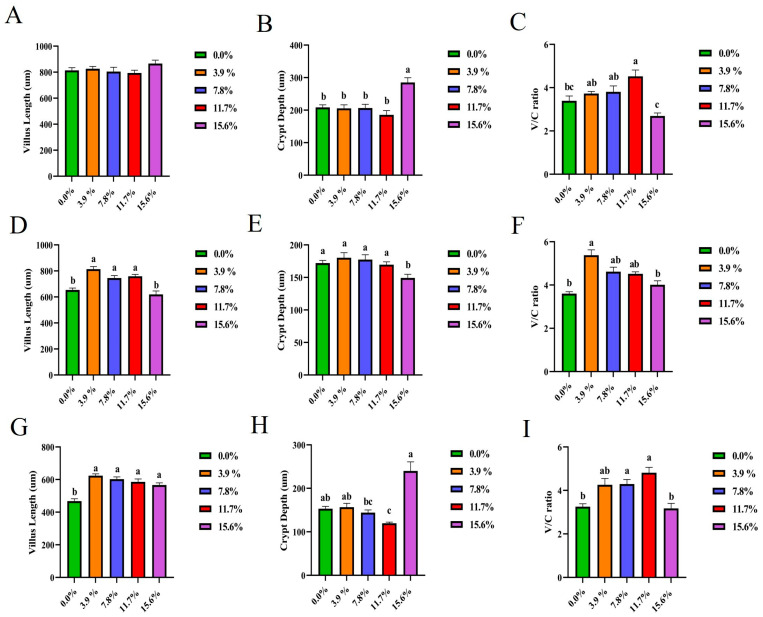
Effect of replacing soybean meal with BSF meal on intestinal villi and crypts in Xichuan black-bone chicken. (**A**) Duodenal villus height; (**B**) Duodenal crypt depth; (**C**) Duodenal V/C; (**D**) Jejunal villus height; (**E**) Jejunal crypt depth; (**F**) Jejunal V/C; (**G**) Ileal villus height; (**H**) Ileal crypt depth; (**I**) Ileal V/C. In x-axis, the 0.0% represents the control group. The small letters a, b, etc., on the top of each bar represent statistical significance. Data with different small letters on each bar are statistically significant (*p* < 0.05), while data with the same letters are not statistically significant (*p* > 0.05).

Compared with the control group, the addition of 11.7% BSF meal significantly increased (*p* < 0.05) the duodenal V/C ratio (Figure 3C) and improved duodenal villus integrity. Representative sections are shown in Figure S1. The addition of 3.9%, 7.8%, and 11.7% BSF meal significantly increased (*p* < 0.05) jejunal villus length (Figure 3D). Adding 3.9% BSF meal significantly increased (*p* < 0.05) the jejunal V/C ratio (Figure 3F) and improved jejunal villus integrity. Representative sections are shown in Figure S1. The addition of 3.9%, 7.8%, 11.7%, and 15.6% BSF meal significantly increased (*p* < 0.05) ileal villus length (Figure 3G), while 11.7% significantly reduced (*p* < 0.05) ileal crypt depth (Figure 3H). Specifically, supplementation with 7.8% and 11.7% significantly increased (*p* < 0.05) the villus-crypt ratio in the ileum (Figure 3I) and improved ileal villus integrity. Representative sections are shown in Figure S1. Consequently, the most pronounced ameliorative effect was observed at the 11.7% supplementation level.

### 3.3. Effects of Black Soldier Fly Larvae Meal at Different Concentrations on Intestinal Tight Junction Proteins

**Figure 4:**
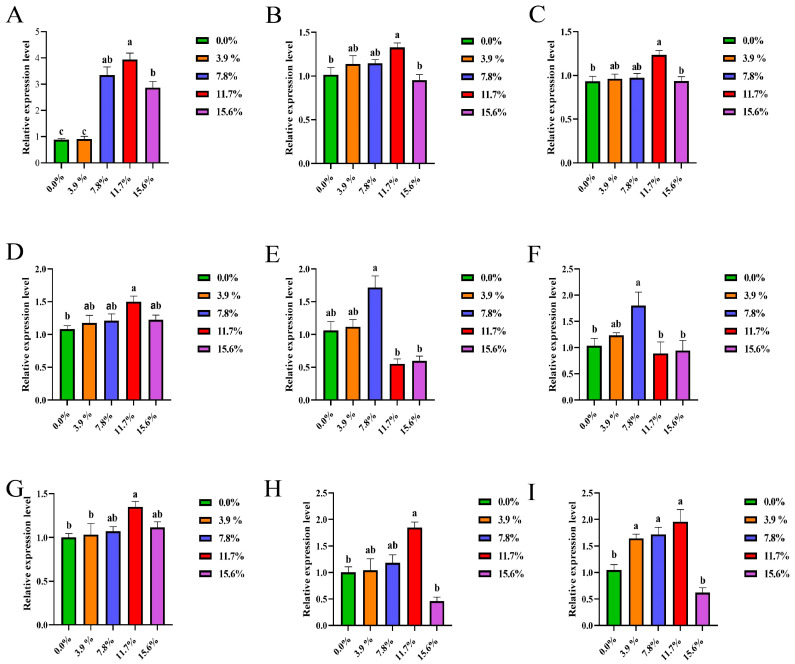
Effect of replacing soybean meal with BSF meal on intestinal tight junction protein genes in Xichuan black-bone chicken. (**A**) Relative expression level of *Claudin-1* genes in the duodenal; (**B**) Relative expression level of *Occludin* genes in the duodenal; (**C**) Relative expression level of *E-cadherin* genes in the duodenal; (**D**) Relative expression level of *Claudin-1* genes in the jejunal; (**E**) Relative expression level of *Occludin* genes in the jejunal; (**F**) Relative expression level of *E-cadherin* genes in the jejunal; (**G**) Relative expression level of *Claudin-1* genes in the ileal; (**H**) Relative expression level of *Occludin* genes in the ileal; (**I**) Relative expression level of *E-cadherin* genes in the ileal. In x-axis, the 0.0% represents the control group. The small letters a, b, etc., on the top of each bar represent statistical significance. Data with different small letters on each bar are statistically significant (*p* < 0.05), while data with the same letters are not statistically significant (*p* ≥ 0.05).

Results demonstrated that supplementation with 7.8%, 11.7%, and 15.6% BSF significantly increased (*p* < 0.05) duodenal *Claudin-1* gene expression (Figure 4A). Supplementation with 11.7% BSF significantly increased (*p* < 0.05) duodenal *Occludin* and *E-cadherin* (Figure 4B,C), jejunal *Claudin-1* (Figure 4D), ileal *Claudin-1*, and *Occludin* gene expression (Figure 4G,H). The addition of 7.8% BSF significantly increased (*p* < 0.05) the gene expression of *E-cadherin* in the jejunum (Figure 4F); the addition of 3.9%, 7.8%, and 11.7% BSF significantly increased (*p* < 0.05) the gene expression of *E-cadherin* in the ileum (Figure 4I).

### 3.4. Effects of Adding 11.7% Black Soldier Fly Larvae Meal on the Gut Microbiota of Xichuan Black-Bone Chickens

**Figure 5:**
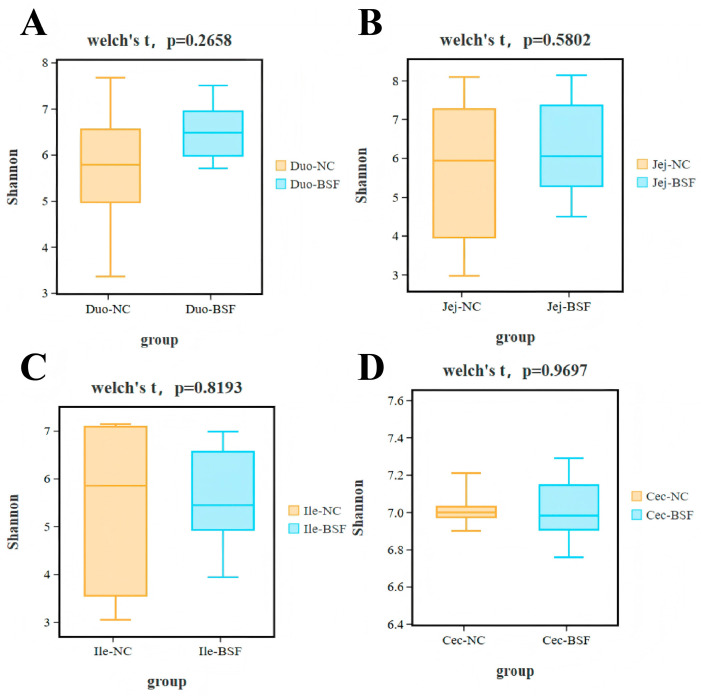
Box plot of α-diversity statistical test results. (**A**) Duodenal Shannon index; (**B**) Jejunal Shannon index; (**C**) Ileal Shannon index; (**D**) Cecal Shannon index.

**Figure 6:**
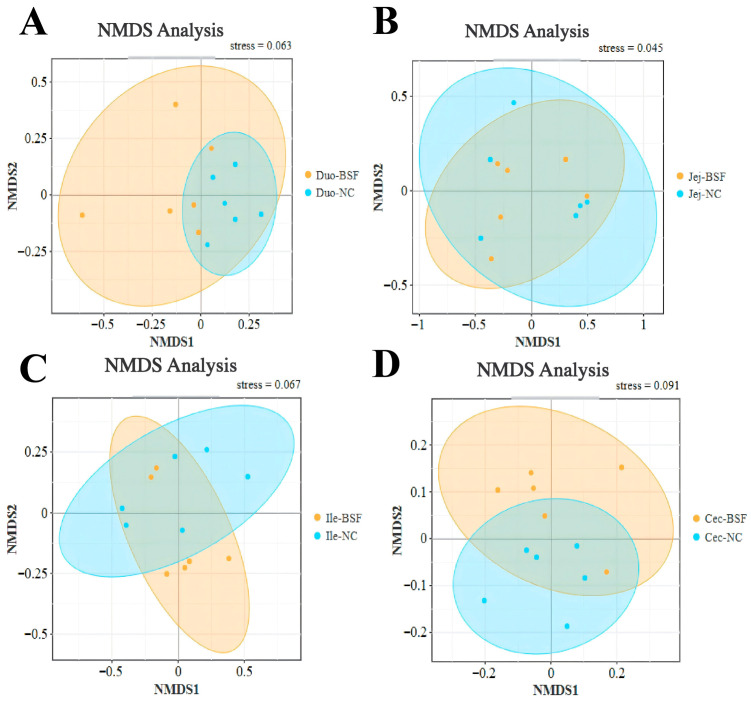
NMDS plot based on Bray–Curtis Distance of OUT levels between the treatment group and the control group. (**A**) Duodenum NMDS; (**B**) Jejunal NMDS; (**C**) Ileal NMDS; (**D**) Cecal NMDS.

**Figure 7:**
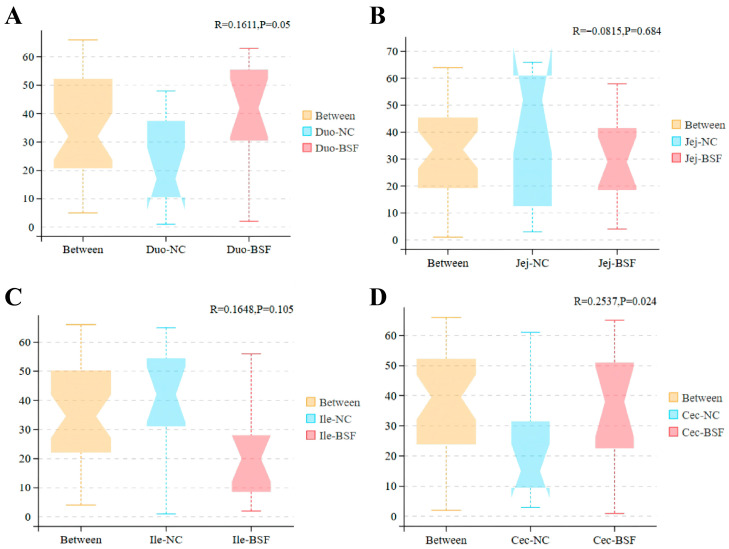
Boxplot of Anosim analysis based on Bray–Curtis Distance of OUT levels between the treatment group and the control group. (**A**) Result of Anosim tests in the duodenum; (**B**) Result of Anosim tests in the jejunal; (**C**) Result of Anosim tests in the ileum; (**D**) Result of Anosim tests in the cecal.

**Figure 8:**
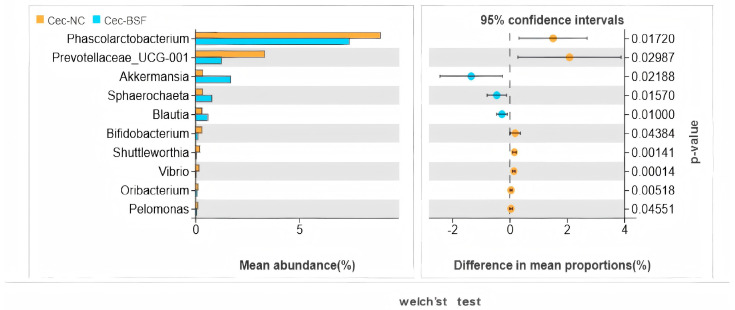
The differential genus level microbiota in the cecum between the supplemented group and the control group.

Compared with the control group, adding 11.7% BSF meal to replace soybean meal numerically increased the Shannon index in the duodenum and jejunum (Figure 5A,B). The Shannon index in the ileum and cecum showed a slight decrease (Figure 5C,D), with the cecum exhibiting the highest Shannon index. Based on the NMDS plot at the OTU level using Bray distance, no significant differences in community distribution were observed in the duodenum, jejunum, and ileum of the 11.7% BSF meal group compared to the control group, whereas the cecum exhibited more pronounced differences (Figure 6). Based on Anosim test results at the OTU level using Bray distance, compared with the control group, the microbial distribution in the duodenum, jejunum, and ileum did not reach statistical significance in the 11.7% BSF meal group, whereas the cecum exhibited significant differences (R = 0.2537, *p* = 0.024) (Figure 7). Therefore, the cecum warrants further attention. Metagenomic sequencing results from the cecum indicate that the species-level NMDS plot revealed a marked difference in cecal microbial distribution between the 11.7% BSF meal group and the control group (Figure 8).

Following Welch’s *t*-test, some cecal intestinal microbiota exhibited significant changes at the genus level. After replacing 11.7% soybean meal with BSF meal, primarily comprising *Akkermansia*, *Sphaerochaeta*, and *Blautia*. The microbiota that significantly decreased mainly comprised *Phascolarctobacterium*, *Prevotellaceae_UCG_001*, *Bifidobacterium*, *Shuttleworthia, Vibrio*, *Oribacteriu m*, and *Pelomonas* (Figure 8).

**Figure 9:**
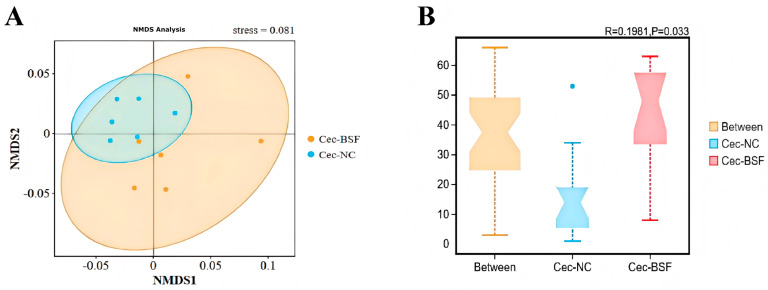
Differences in microbial distribution and differential microbiota at the species level between the cecal supplementation group and the control group. (**A**) Cecal NMDS; (**B**) Result of Anosim tests in the cecal.

In the further validation of β-diversity between the control group and the 11.7% supplementation group via microbial metagenomics, the species-level Anosim test results indicated that, compared with the control group, the bacterial distribution in the cecum of the supplemented group showed a significant difference (R = 0.1981, *p* = 0.033) (Figure 9).

### 3.5. Effects of Adding 11.7% Black Soldier Fly Larvae Meal on Metabolites in the Cecum of Xichuan Black-Bone Chickens

**Figure 10:**
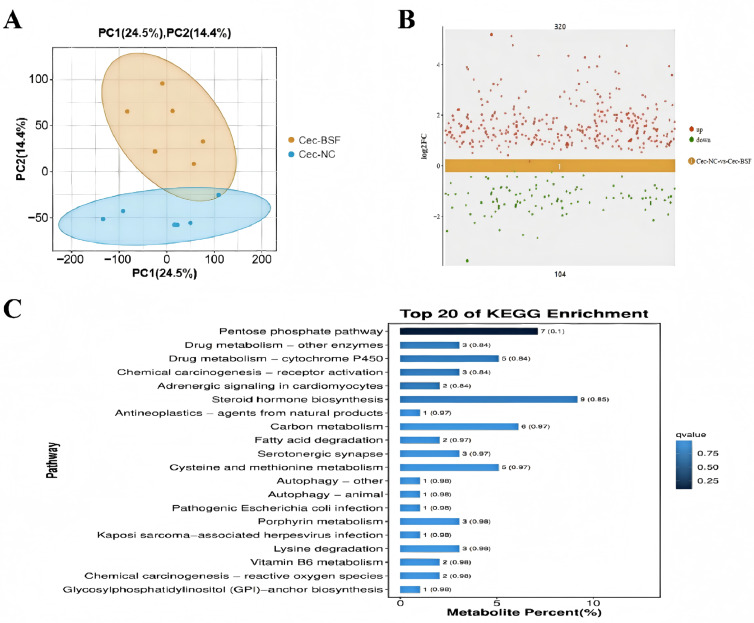
Analysis of the main components of metabolites in the 11.7% addition group and the control group, as well as KEGG enrichment analysis of differential metabolites. (**A**) Cecal metabolite PCA plot; (**B**) Scatter plot of differences between the control group and the supplemented group; (**C**) KEGG enrichment analysis of differential metabolites.

Principal component analysis of cecal metabolites between the control group and the group supplemented with 11.7% BSF meal revealed distinct separation on the PCA coordinate plot, indicating that replacing soybean meal with 11.7% BSF larvae meal significantly influenced cecal metabolites (Figure 10A). Analysis of differentially expressed metabolites revealed that, compared to the control group, the 11.7% BSF larvae meal group exhibited 320 significantly upregulated metabolites and 104 significantly downregulated metabolites (Figure 10B). These differentially expressed metabolites were enriched in pathways including the pentose phosphate pathway, drug metabolism-other enzymes, drug metabolism-cytochrome P450, chemical carcinogenesis-receptor activation, and adrenergic signaling in cardiomyocytes pathways (Figure 10C). These pathways are primarily associated with immunity and metabolism, warranting attention for their potential correlation with immune indicators.

### 3.6. Correlation of Microorganisms, Metabolites, and Immune Markers

**Figure 11:**
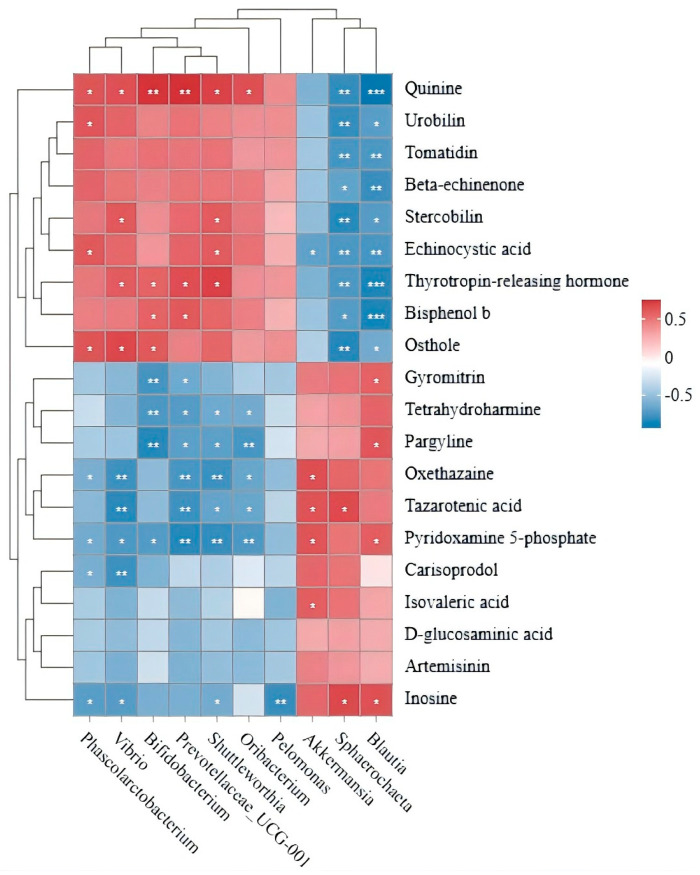
Association analysis between differential microbiota and differential metabolites. Correlation heat map of genus level bacteria and metabolites. * indicates *p* < 0.05 in the correlation test, ** indicates *p* < 0.01, *** indicates *p* < 0.001.

In the correlation analysis of signature differential bacteria and metabolites in the cecum, the inter-group differential metabolites between the control group and the 11.7% BSF meal supplement group were ranked by abundance. Among the top 20 metabolites in descending order of abundance, isovaleric acid, D-glucosaminic acid, inosine, tazarotenic acid, and artemisinin are associated with immune function or gut health and are designated as key metabolites (Table 4). At the genus level, only 10 distinct microbial species were identified in the cecum between the control group and the 11.7% BSF meal supplement group. The top 10 species by abundance are listed in Table 5. Among genus-level differential bacteria, *Akkermansia* showed a significant positive correlation (*p* < 0.05) with isovaleric acid; *Sphaerochaeta* and *Blautia* correlated positively (*p* < 0.05) with inosine; while *Akkermansia* and *Sphaerochaeta* correlated positively (*p* < 0.05) with talazoparib. (Figure 11).

**Figure 12:**
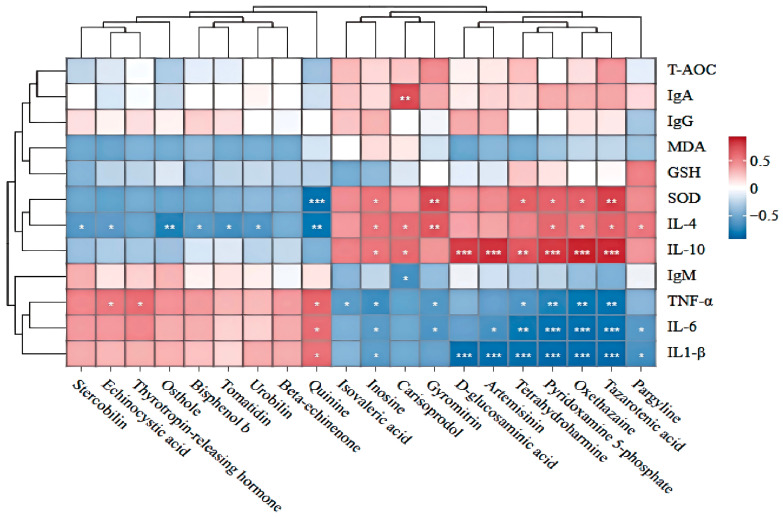
Association analysis between differential metabolites and phenotypic indicators. * indicates *p* < 0.05 in the correlation test, ** indicates *p* < 0.01, *** indicates *p* < 0.001.

**Figure 13:**
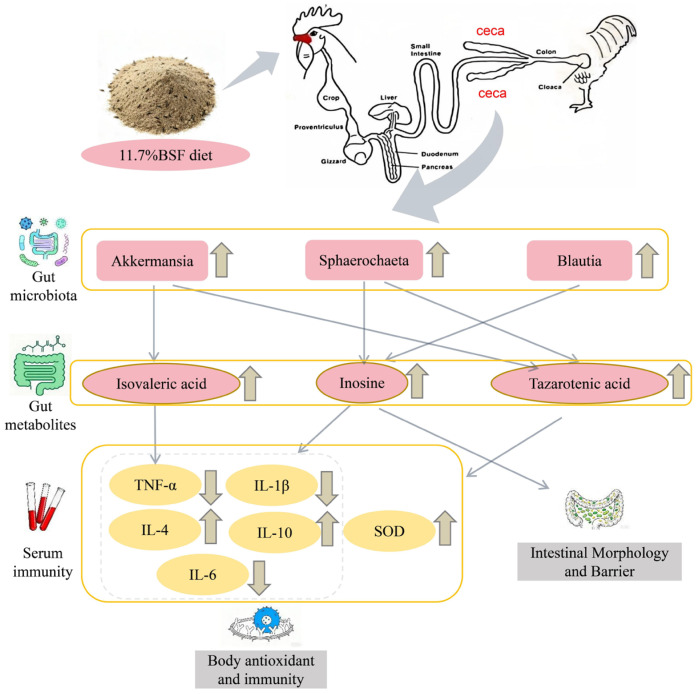
The cascade regulation of black soldier fly on the antioxidant indicators, immune system, and intestinal health of the organism.

In the correlation analysis between differential metabolites in the cecum and differential immune markers in the body, isovaleric acid showed a significant negative correlation (*p* < 0.05) with TNF-α; D-glucosamine exhibited a significant positive correlation (*p* < 0.05) with IL-10 and a significant negative correlation (*p* < 0.05) with IL-1β; inosine showed significant positive correlations (*p* < 0.05) with SOD, IL-4, and IL-10, and significant negative correlations (*p* < 0.05) with TNF-α, IL-6, and IL-1β; tazalotide exhibited significant positive correlations (*p* < 0.05) with SOD, IL-4, and IL-10, and significant negative correlations (*p* < 0.05) with TNF-α, IL-6, and IL-1β; artemisinin showed a significant positive correlation with IL-10 and a significant negative correlation (*p* < 0.05) with IL-6 and IL-1β (Figure 12). Potential cascading regulatory mechanisms may exist among gut microbiota, metabolites, and epigenetic markers, as illustrated in Figure 13.

## 4. Discussion

Soybean meal is currently facing the dual limitations of resource supply shortage and the intensification of environmental pollution during the breeding process. This issue has become one of the key factors restricting the sustainable development of the feed industry [17]. Meanwhile, older breeding roosters commonly exhibit reduced antioxidant capacity and immune function, alongside diminished intestinal function. This problem directly affects the reproductive performance and genetic stability of the breeding chicken population [18]. It is worth noting that BSF protein, as a balanced animal protein source in terms of nutritional composition, has great potential in improving antioxidant and immune-related indicators of poultry, regulating the balance of intestinal microecology, and enhancing the level of intestinal health. This study aims to systematically evaluate the effects of replacing soybean meal with black soldier fly larvae meal at varying ratios on the antioxidant capacity, immune parameters, and intestinal physiological morphology of Xichuan black-bone chickens. It seeks to identify the optimal supplementation ratio and predict the cascading regulatory pathway of BSF via the ‘feed–gut microbiota–gut metabolites–phenotypic indicators’ pathway.

Antioxidant capacity is an important indicator for evaluating the body’s resistance to free radical damage. It is closely related to the process of cell differentiation and tissue development. Maintaining a relatively balanced antioxidant system plays a key role in the body’s immune function [19]. This experiment found that adding 11.7% and 15.6% black soldier fly larval powder could significantly enhance the T-AOC, SOD, and GSH in the serum of Xichuan black-bone chickens. Among them, the improvement in the activity of serum T-AOC and SOD reached a significant level (*p* < 0.05). T-AOC can effectively reduce the level of oxidative stress by eliminating free radicals, and protect cell membranes, proteins, and DNA from oxidative damage [20]. Poultry with strong antioxidant capabilities usually exhibit better immune performance. They can not only effectively resist pathogen invasion, but also reduce the inflammatory response caused by stress [21]. SOD can maintain normal cellular functions by eliminating superoxide anion free radicals, and it is involved in immune regulation and tissue repair [22]. The core function of serum GSH lies in its participation in the body’s antioxidant defense and metabolic regulation, serving as a key substance for maintaining serum redox equilibrium [23]. Consequently, the addition of 11.7% and 15.6% BSF significantly enhanced the antioxidant capacity of Xichuan black-bone chickens.

Chicken serum immunoglobulin, as the core effector molecule of avian humoral immunity, mainly includes three types: IgA, IgG, and IgM. Each of these plays a significant role in mucosal immunity, systemic immunity, and the early immune response. This experiment found that compared with the control group, adding 11.7% BSF powder could significantly increase the levels of serum IgA and IgM (*p* < 0.05), and also showed an improving trend for serum IgG levels. IgA mainly exists in monomer and dimer forms, among which secretory IgA (SIgA) is the main effector molecule of mucosal immunity and plays a key role in immune defense at mucosal surfaces such as the respiratory tract and digestive tract. Serum IgA levels can reflect the mucosal immune status of the chicken flock [24,25]. IgG is the main antibody that mediates humoral immunity [26]. IgM has multiple antigen-binding sites and can efficiently neutralize pathogens and activate the complement system [27]. Studies have shown that feeding probiotics can significantly increase the levels of IgA and IgM in the serum of mice, thereby enhancing the immune function of the body [28]. Similarly, BSF powder as a substitute for soybean meal can promote the immune function of poultry. In the complex physiological process of the inflammatory response, anti-inflammatory factors and pro-inflammatory factors play a crucial regulatory role. Both are essential for maintaining the health of the body. Adding different proportions of BSF powder can significantly increase the concentrations of anti-inflammatory factors IL-4 and IL-10 in the serum, and significantly reduce the concentration of the pro-inflammatory factor IL-1β in the serum. Adding 7.8%, 11.7%, and 15.6% BSF powder can significantly reduce the concentrations of pro-inflammatory factors TNF-α and IL-6 in the serum. Among them, adding 11.7% has the most significant effect. IL-4 can inhibit the activation of Th1 cells, reduce the secretion of pro-inflammatory factors, and promote the production of IgE by B cells, thereby regulating humoral immunity and exerting anti-inflammatory effects in inflammatory conditions such as allergic reactions [29]. IL-10 can inhibit the production of pro-inflammatory factors by mononuclear macrophages, reduce the activity of immune cells, inhibit the inflammatory signaling pathways, and effectively alleviate the damage caused by inflammatory responses to the body [30]. TNF-α, IL-6, and IL-1 can be rapidly activated during the initial stage of inflammation. TNF-α can induce cell apoptosis, activate immune cells, and trigger an inflammatory cascade reaction, thereby prompting the release of other pro-inflammatory factors [31]. IL-1β can stimulate T cell activation and B cell proliferation, playing a crucial role in acute inflammatory responses. It works in conjunction with TNF-α to amplify the inflammatory signal [32,33]. IL-6 is involved in immune regulation, the acute-phase response, raising body temperature, promoting the synthesis of acute-phase proteins in the liver, and exacerbating inflammatory damage [34,35]. Antibacterial peptides (such as attacin type) can significantly increase the content of IL-10 in chicken serum [36]. Chitin can be degraded by intestinal microorganisms into chitosan oligosaccharides, which can block key pro-inflammatory signaling pathways and induce anti-inflammatory factors such as IL-10, thereby reducing the transcription and synthesis of pro-inflammatory factors, regulating the activity of immune cells, and enhancing the intestinal barrier function [37]. This might be due to the presence of substances such as chitin and antibacterial peptides in the 11.7% BSF feed, which enhance the anti-inflammatory factors and reduce the pro-inflammatory factors in Xichuan black-bone chickens [38]. The chicken spleen, as an important peripheral immune organ of the immune system, plays a crucial role in various physiological processes such as immune defense, blood filtration, hematopoiesis, blood storage, and antibody production. It is one of the core organs that maintain the immune homeostasis of the body [39]. This study found that adding 7.8% and 11.7% BSF powder could significantly improve the integrity of the white pulp morphology of the spleen, and the boundary between the red pulp and white pulp was also clear. In histology, the morphological structure of the white pulp and red pulp, as well as the clarity of their boundary, are important indicators for evaluating the immune function of the spleen. The immune function of the spleen is crucial in the body’s immunity [40,41], suggesting that BSF has the potential to enhance the immune function of the body.

The length of intestinal villi and the depth of crypts in the small intestine are important morphological indicators for evaluating the digestive and absorptive functions of the small intestine as well as intestinal health. The intestinal villi, as the finger-like protrusions of the intestinal mucosal epithelium into the intestinal lumen, have a positive correlation with the absorptive surface area of the small intestine. Longer villi structures can significantly increase the contact area between the intestinal lumen and nutrients. The crypts serve as the proliferation areas of intestinal epithelial cells; their depth reflects the renewal rate of intestinal epithelial cells [42]. There is a dynamic balance between them. The ratio of crypt-to-cone (V/C) is usually regarded as an important indicator for evaluating intestinal physiological function. When this ratio increases, the digestive and absorptive capacity improves; when it decreases, the digestive and absorptive capacity decreases. At the same time, the intestinal barrier may be damaged. Nutritional factors have a significant impact on the villi and crypts of the small intestine [43]. Adding 11.7% BSF powder can significantly increase the villus ratio of the duodenum and ileum, increase the length of the jejunal and ileal villi, and reduce the depth of the ileal crypts. Research shows that a high-quality feed formula can promote the development of small intestinal villi, maintain an appropriate crypt depth, and thereby ensure intestinal health [44]. Therefore, 11.7% BSF is suitable for use in the daily diet of Xichuan black-bone chickens. Intestinal junction proteins play a central role in maintaining the barrier function of the intestine. They not only prevent the invasion of harmful substances but also participate in regulating the functions of intestinal immune cells and the balance of the intestinal microecology. In terms of immune regulation, the expression of junction proteins is related to the migration and activation of immune cells [45]. Adding 11.7% BSF powder can significantly increase the gene expression levels of *Claudin-1*, *Occludin,* and *E-cadherin* in the duodenum and ileum; it also significantly increases the gene expression level of *Claudin-1* in the jejunum. *Occludin* and members of the *Claudin* family interact through special transmembrane domains to form a tight barrier, effectively preventing the leakage of pathogens, harmful substances, and large molecules in the intestinal tract, which is crucial for maintaining the selective permeability function of the intestine [46,47]. *E-Cadherin* mainly mediates the adhesion between cells. By connecting with the actin cytoskeleton, it maintains the normal arrangement and structural integrity of intestinal epithelial cells, providing a structural basis for the normal physiological functions of the small intestine [48]. Consistent with the findings of studies on supplementing BSF in broiler diets, in Xichuan black-bone chickens, supplementation with 15.6% BSF larval meal led to a decrease in the intestinal villus-to-crypt ratio and junction protein content compared with the 11.7% supplementation group, which was attributed to the excess effect [49]. Therefore, adding 11.7% BSF powder can improve the intestinal health of Xichuan black-bone chickens.

The intestinal microbiota plays a significant role in maintaining the health and production performance of poultry. They are involved in the digestion of feed and the absorption of nutrients, breaking down indigestible substances such as cellulose, and producing beneficial metabolites such as short-chain fatty acids [50]. Moreover, the intestinal microbiota can maintain the intestinal barrier function by regulating the immune system and inhibiting the growth of pathogenic bacteria, thereby enhancing the host’s immunity. The homeostasis of the intestinal microbiota is crucial for the health of poultry [51]. This is an important research method for predicting the impact of feed on the body of poultry through the changes in intestinal microbial indicators in poultry. The α-diversity of intestinal microorganisms is mainly used to measure the richness and evenness of microorganisms in a specific ecosystem (such as the intestine). Studies have shown that a higher Shannon index is usually associated with a more stable microbial community and better intestinal health status [52]. Adding 11.7% of BSF numerically increased the Shannon index of the duodenum and jejunum, while slightly reducing that of the ileum and cecum; this is related to intestinal stability. It is noteworthy that the Shannon index of the cecum is higher than that of the duodenum, jejunum, and ileum, indicating that the richness and uniformity of the microbiota in the cecum are higher, and thus having greater research significance. The β-diversity of intestinal microbiota is often used to compare the differences in the composition of intestinal microbiota communities among different samples such as different individual animals, animal groups in different breeding environments, etc. It can reflect the changes in species composition and reveal the degree of variation in the microbial community structure among different ecological niches. The test results indicate that after adding 11.7% BSF powder, significant changes occurred in the cecal microorganisms, but there was no significant impact on the distribution of intestinal microorganisms in the duodenum, jejunum, and ileum. After examination, the addition of 11.7% BSF powder did not reach a significant level of influence on the distribution of intestinal microorganisms in the small intestine of Xichuan black-bone chickens, but it did have a significant impact on the distribution of the cecal intestinal flora, which may also simultaneously affect the functional aspects of the cecal intestinal flora. Therefore, the path by which BSF powder exerts its effects on the body through cecal microorganisms is worthy of further attention.

In the analysis of microbial communities, the genus level is one of the commonly used classification levels when conducting community composition analysis, diversity assessment, and functional correlation analysis. The genera with significantly enhanced bacterial populations in the cecum at the genus level include *Akkermansia*, *Sphaerochaeta*, and *Blautia*, while the genera with significantly reduced populations are *Phascolarctobacterium* and *Prevotellaceae_UCG_001*. *Akkermansia* can produce short-chain fatty acids, which have beneficial effects on the intestinal tract and the immune system of the body [53]. *Sphaerochaeta* is associated with fat deposition [54]. *Blautia* can produce short-chain fatty acids, enhance immunity, and alleviate inflammatory and metabolic diseases [55,56]. *Phascolarctobacterium* is an obligate anaerobic and Gram-negative bacterium that can produce short-chain fatty acids, including acetate and propionate, and may be related to the host’s metabolic state and mood [57]. *Prevotellaceae_UCG-001* can degrade cellulose and xylan, and exert the effect of generating short-chain fatty acids [56]. These results indicate that adding 11.7% BSF powder can increase the content of most beneficial bacteria in the cecum and reduce the content of harmful bacteria. Although it significantly reduced the abundance of beneficial bacteria such as *Phascolarctobacterium* in the cecum, it may have improved the body’s immunity and intestinal health by increasing the abundance of beneficial bacteria such as *Akkermansia* and *Blautia* in the cecum. As a more detailed classification unit within the microbial classification system, the species level is a crucial analytical level for conducting more in-depth and precise analysis of microbial groups. The NMDS graph based on the species level clearly shows that compared with the control group, the distribution of cecal microorganisms in the 11.7% BSF powder addition group has significant differences at the species level. After the Anosim test analysis, *p* < 0.05, indicating that the distribution of cecal microorganisms in the two groups at the species level has a significant difference. This is consistent with the test results based on the OUT level in 16s rRNA sequencing, and is complementary verification, further confirming the impact of replacing soybean meal with 11.7% BSF powder on the cecal microbial flora.

The intestinal metabolites of poultry are an important medium for the interaction between intestinal microorganisms and the host [58]. Their production is closely related to the fermentation activities of intestinal microorganisms. Intestinal microorganisms decompose complex carbohydrates, proteins, and other substances in the feed to produce short-chain fatty acids (such as acetic acid, propionic acid, and butyric acid), amino acid metabolites (such as ammonia and polyamines) and vitamins, etc. [59]. These metabolites can provide energy for the animal body, regulate inflammatory factors, participate in regulating the pH value of the intestinal tract, maintain the intestinal barrier function, and play an important role in the host’s immune regulation [60]. In addition, intestinal metabolites are closely related to antioxidant-related indicators. Some substances produced by intestinal microorganisms (such as glutathione and polyphenolic compounds) have direct antioxidant effects, which can eliminate free radicals and alleviate oxidative stress [61]. Therefore, poultry intestinal metabolites play a key role in regulating the interaction between intestinal microorganisms and the host, and are of great significance in regulating the antioxidant indicators, immune system, and intestinal health of the organism. Principal Component Analysis (PCA) is a method for reducing dimensions to display the overall differences among samples. If the samples of the experimental group and the control group are clearly separated in the PCA graph, it indicates that there are significant differences in metabolites between the two groups [62]. From the PCA graph, it can be seen that the cecum metabolites of the 11.7% BSF powder addition group and the control group can be clearly separated, indicating that the addition of 11.7% BSF powder has a significant impact on the cecum metabolites of Xichuan black-bone chickens. KEGG pathway enrichment analysis is commonly used in the analysis of metabolomic or transcriptomic results, and is used to analyze the functions of metabolites or genes. Compared with the control group, after adding 11.7% BSF powder, 320 metabolites in the cecum of Xichuan black-bone chickens showed significant upregulation, while 104 showed significant downregulation. These metabolites were mainly enriched in the pentose phosphate pathway, drug metabolism-other enzymes, drug metabolism-cytochrome P450, chemical carcinogenesis- receptor activation, and the adrenergic signaling pathway in cardiac muscle cells. The pentose phosphate pathway is not only an important branch of glucose metabolism but also plays a key role in inhibiting oxidative stress. The NADPH derived from PPP also supports the purposeful production of reactive oxygen species (ROS) and reactive nitrogen species (RNS) by cells for signal transduction and killing pathogens, thereby exerting an immune resistance effect [63]. In the drug metabolism-other enzymes and drug metabolism-cytochrome P450 pathways which are related to drug metabolism [64], and the receptor activation pathway of chemical carcinogenesis is mainly associated with the binding of certain chemical carcinogens to specific receptors within cells [65]. The adrenergic signaling pathway in cardiac muscle cells refers to the process where adrenaline and noradrenaline, through the activation of adrenergic receptors (Adrenergic Receptors, ARs) on cardiac muscle cells, trigger a series of intracellular signal transduction, ultimately regulating cardiac function [66]. Therefore, the changes in metabolites after adding 11.7% BSF powder are worthy of attention in terms of their relationship with the body’s immunity.

The intestinal microbiota can produce various metabolites through the metabolism of food and endogenous substances in the host. These metabolites not only affect the host’s metabolic health but are also closely related to the occurrence of various diseases. Some metabolites can affect the intestinal barrier, and can also influence serum immune indicators through entering the bloodstream or other indirect means [67]. At the same time, serum immune indicators can also reflect the immune status of local organs (such as the intestine) [68]. There is a positive correlation between the microbiota and the metabolites it produces. Therefore, after conducting a correlation analysis of the metabolites related to the intestinal microbiota, the significant positively correlated microbiota and metabolites deserve attention. The abundance of metabolites determines the degree of impact on the animal body. After correlating the high-content differential metabolites with differential microbiota, at the genus level, the bacteria significantly positively correlated with isovaleric acid were *Akkermansia*. Meanwhile, isovaleric acid was significantly negatively correlated with the pro-inflammatory factor TNF-α in the immune indicators. *Akkermansia* can promote the production of short-chain fatty acids, and isovaleric acid is one type of short-chain fatty acid [69,70]. Studies have shown that valerate can promote the secretion of anti-inflammatory factors (such as IL-10) by regulating lymphocytes, thereby exerting a regulatory effect on the immune system of the body [71,72]. By analogy, adding 11.7% BSF powder may regulate the metabolite isovaleric acid in the cecum by regulating *Akkermansia*, thereby reducing the content of the pro-inflammatory factor TNF-α, and thus improving the immune function of the body. The bacteria that have a significant positive correlation with inosine are *Sphaerochaeta* and *Blautia.* At the same time, inosine has a significant negative correlation with pro-inflammatory factors IL-1β, IL-6, and TNF-α, and a significant positive correlation with anti-inflammatory factors IL-4 and IL-10 as well as the antioxidant indicator SOD. Studies have shown that inosine (Inosine) is a naturally occurring nucleoside compound that can regulate energy metabolism, protect cell functions, and promote tissue repair. It can inhibit the production of pro-inflammatory factors such as TNF-α, IL-6, and IL-1β, promote the production of anti-inflammatory factors such as IL-4 and IL-10 [73,74], and improve the intestinal mucosal barrier. It can prevent and inhibit intestinal inflammation through the PPARγ pathway [75]. Therefore, it can be predicted that adding 11.7% BSF powder as a substitute for soybean meal can affect the intestinal metabolite inosine through *Sphaerochaeta* and *Blautia*, thereby regulating inflammatory factors, increasing the body’s antioxidant levels, and improving the intestinal barrier. The bacteria that have a significant positive correlation with tazarotene are *Akkermansia* and *Sphaerochaeta*. At the same time, tazarotene has a significant negative correlation with pro-inflammatory factors such as TNF-α, IL-1β, and IL-6, and a significant positive correlation with serum SOD, IL-4, and IL-10. Studies have shown that tazarotene can inhibit the production of IL-1 and TNF-α [76]. The results are similar. It is speculated that *Akkermansia* and *Sphaerochaeta* play a positive regulatory role in tazarotene, thereby improving the immune and antioxidant indicators of the body. Therefore, *Akkermansia*, *Sphaerochaeta*, and *Blautia* may improve the body’s immunity through intestinal metabolites such as isovaleric acid, inosine, and tazarotene. *Sphaerochaeta* and *Blautia* may also improve intestinal health through inosine. The metabolites or other products in the intestine can directly or indirectly affect the intestinal barrier and serum immune indicators [77]. By correlating different bacteria, different metabolites, intestinal health, and immune indicators of the body, it was found that there are significant correlations at the species level between different bacteria and different metabolites. This verifies that adding 11.7% BSF powder may have a significant impact on the cecal microbiota of Xichuan black-bone chickens, thereby having a significant impact on the body’s antioxidant capacity, immunity, and intestinal health.

## 5. Conclusions

This study investigated the impact approaches of adding different proportions of BSF powder as a substitute for soybean meal on the antioxidant defense, immune system, and intestinal health of Xichuan black-bone chickens. By adding 3.9%, 7.8%, 11.7%, and 15.6% BSF powder to replace soybean meal, it was found that 11.7% was the optimal addition ratio. The intestinal microbiota of the optimal addition group was tested. It was found that adding 11.7% BSF powder as a substitute for soybean meal had a significant impact on the distribution of the microbiota in the cecum and changed the distribution of metabolite profiles in the cecum of Xichuan black-bone chickens. Further correlation analysis was conducted on the microorganisms and metabolites of the cecum. Adding 11.7% BSF meal as a substitute for soybean meal could regulate metabolites such as isovaleric acid, inosine, and tazarotene through *Akkermansia*, *Sphaerochaeta*, and *Blautia* to improve the body’s immunity. By regulating the metabolite inosine through *Sphaerochaeta* and *Blautia*, it could also improve intestinal health. This study provides new insights and theoretical support for soybean meal substitution and the antibiotic ban.

## Figures and Tables

**Table 1:** Main nutritional components of black soldier fly.

Component	Content
CP	44.61%
Met	2.04%
Lys	3.21%
Ca	0.017%
P	0.01%
ME(MJ/kg)	16.83

**Table 2:** Composition and nutrient levels of diets with different substitution ratios (air-dry basis) %.

Ingredient	0.0%	3.9%	7.8%	11.7%	15.6%
Maize	53	53	53	53	53
Soybean Meal	15.6	11.7	7.8	3.9	0
BSF	0	3.9	7.8	11.7	15.6
Bran	22.77	23.76	24.74	25.71	26.7
Soybean oil	3.68	2.75	1.83	0.92	0
Mountain Flour	1.57	1.61	1.64	1.67	1.7
Lys	0.14	0.1	0.07	0.04	0
Met	0.24	0.18	0.12	0.06	0
Premix	3	3	3	3	3
Total	100	100	100	100	100
CP	14.82	14.86	14.94	15.01	15.05
ME	11.27	11.27	11.27	11.27	11.27
Lys	0.78	0.78	0.78	0.78	0.78
Met	0.53	0.53	0.53	0.53	0.53
Ca	1.12	1.10	1.11	1.10	1.10
P	0.53	0.50	0.50	0.48	0.47

Note: The nutritional composition of the premix (per kilogram of feed) is as follows: vitamin A 100 KIU, vitamin D3 100 KIU, vitamin E 600 KIU, vitamin K3 100 mg, vitamin B1 50 mg, vitamin B2 166 mg, vitamin B6 117 mg, vitamin B12 0.3 mg, D-biotin 6.7 mg, folic acid 25 mg, pantothenic acid 334 mg, niacin 800 mg, copper 200 mg, iron 2000 mg, manganese 2000 mg, zinc 2000 mg, iodine 20 mg, and selenium 10 mg. The nutritional components of the feed are measured values.

**Table 3:** Primer sets for quantitative real-time fluorescence PCR.

Genes	Primers	Sequences	Primer Size (bp)
*Claudin-1*	F	CATACTCCTGGGTCTGGTTGGT	22
	R	GACAGCCATCCGCATCTTCT	20
*Occludin*	F	ACGGCAGCACCTACCTCAA	19
	R	GGGCGAAGAAGCAGATGAG	19
*E-cadherin*	F	CCTCCAGGATGTGAATGACAACG	23
	R	ATGCTCCAGTGCTGCCTTGAAG	22
*GAPDH*	F	TGCTGCCCAGAACATCATCC	20
	R	ACGGCAGGTCAGGTCAACAA	20

**Table 4:** The top 20 differential metabolites in terms of content.

The Name of the Collection	Metabolite Name
The top 20 differentially expressed metabolites in abundance	Stercobilin, Quinine, **Isovaleric acid**, Gyromitrin, **D-glucosaminic acid**, Urobilin, Osthole, Tetrahydroharmine, Oxethazaine, Pargyline, **Inosine**, Beta-echinenone, Tomatidin, Carisoprodol, Echinocystic acid, **Tazarotenic acid**, Thyrotropin-releasing hormone, Pyridoxamine 5-phosphate, **Artemisinin**

**Table 5:** The top 10 differentially abundant bacterial genera based on relative abundance.

The Name of the Collection	The Name of the Microorganism
The top 10 differentially abundant bacterial genera based on relative abundance (only 10)	*Phascolarctobacterium*, *Prevotellaceae_UCG-001*, *Akkermansia*, *Sphaerochaeta*, *Blautia*, *Bifidobacterium*, *Shuttleworthia*, *Vibrio*, *Oribacterium*, *Pelomonas*

## Data Availability

The data generated in this study are publicly available in the National Center for Biotechnology Information (NCBI) BioProject database. The 16S rRNA gene sequencing data are accessible under accession number PRJNA1391823 (https://dataview.ncbi.nlm.nih.gov/object/PRJNA1391823?reviewer=ec4cva7qrn159q7gbs9nkiro7e, accessed on 21 December 2025), and the metagenomic sequencing data are available under accession number PRJNA1391896 (https://dataview.ncbi.nlm.nih.gov/object/PRJNA1391896?reviewer=9ab9jgfr44rgq8sjsteokm1et, accessed on 22 December 2025).

## Supplementary Materials

The following supporting information can be downloaded at: https://www.mdpi.com/article/10.3390/antiox15040408/s1, Figure S1: The Effects of Different Proportions of BSF Powder on Intestinal Morphology.

## Abbreviations

BSF	Black soldier fly larvae
T-AOC	Total Antioxidant Capacity
SOD	Superoxide Dismutase
GSH	Glutathione
MDA	Malondialdehyde
IgA	Immunoglobulin A
IgG	Immunoglobulin G
IgM	Immunoglobulin M
IL-4	Interleukin-4
IL-10	Interleukin-10
TNF-α	Tumor Necrosis Factor-alpha
IL-1β	Interleukin-1β
IL-6	Interleukin-6
V	Villus Height
C	Crypt Depth
V/C	Villus Height/Crypt Depth
