# Supplementary figures and images for: Effects of Replacing Soybean Meal with Different Proportions of Black Soldier Fly Larvae Meal on Antioxidant Indicators, Immune System, and Gut Health of Xichuan Black-Bone Chickens
Source: Antioxidants (Basel). 2026 Mar 24;15(4):408. doi: 10.3390/antiox15040408 (PMC13113115; doi:10.3390/antiox15040408)

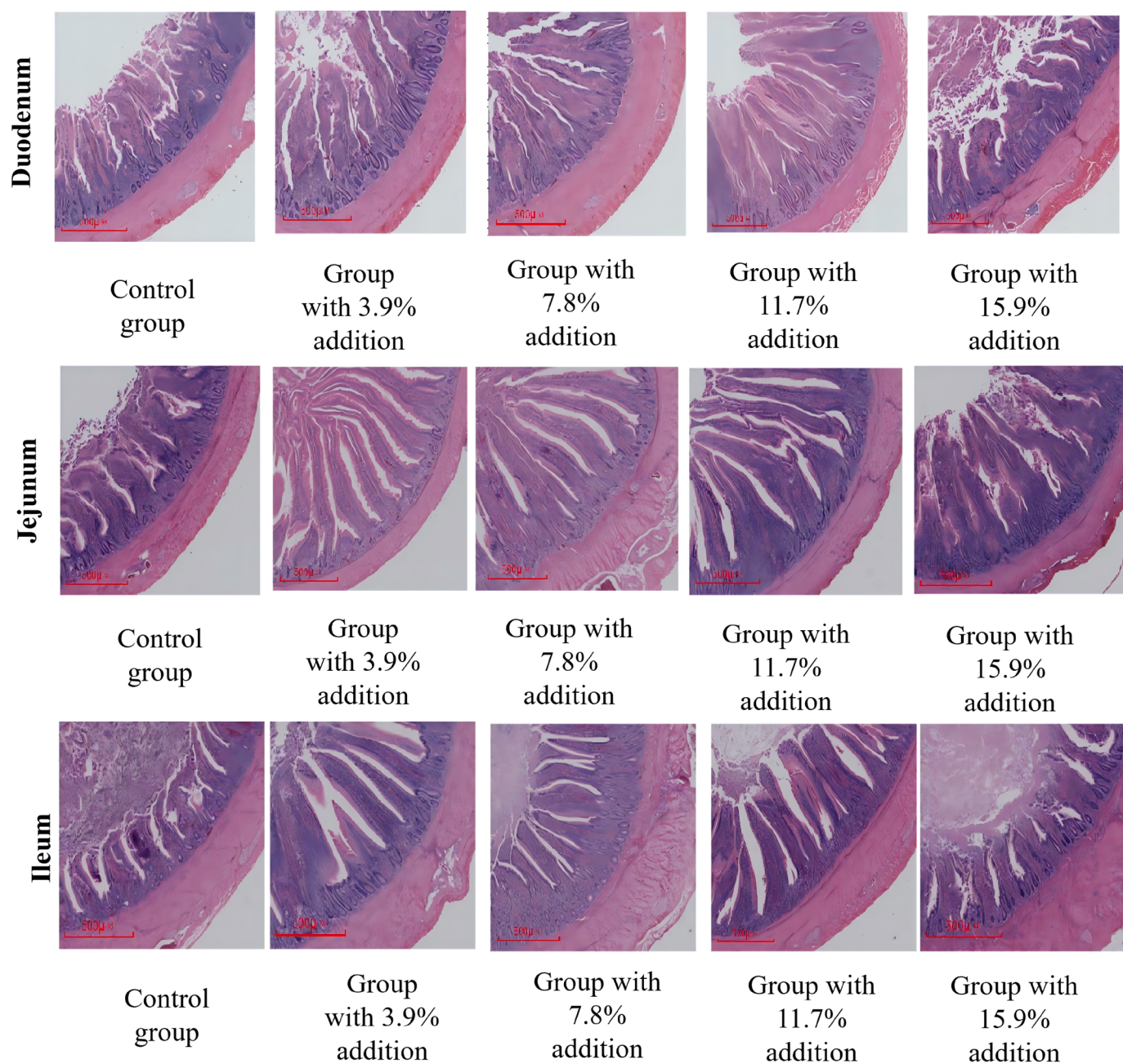

**Figure S1.** The Effects of Different Proportions of BSF Powder on Intestinal Morphology.

Supplement: Supplementary file 1 [file antioxidants-15-00408-s001.zip › antioxidants-4167130-supplementary.pdf]
